# Combining ability and gene action for resistance to Fusarium ear rot in tropical maize hybrids

**DOI:** 10.3389/fpls.2025.1509859

**Published:** 2025-01-30

**Authors:** Stella Bigirwa Ayesiga, Patrick Rubaihayo, John Bosco Sempiira, Emmanuel Amponsah Adjei, Isaac Onziga Dramadri, Bonny Michael Oloka, Julius Pyton Sserumaga

**Affiliations:** ^1^ Department of Crop Science and Horticulture, College of Agriculture and Environmental Sciences, Makerere University, Kampala, Uganda; ^2^ National Livestock Resources Research Institute, National Agricultural Research Organization, Kampala, Uganda; ^3^ Makerere University Regional Centre for Crop Improvement (MaRCCI), College of Agriculture and Environmental Sciences, Makerere University, Kampala, Uganda; ^4^ Council for Scientific and Industrial Research (CSIR)– Savanna Agriculture Research Institute, Tamale, Ghana; ^5^ Department of Horticultural Sciences, North Carolina State University, Raleigh, NC, United States

**Keywords:** Zea mays, Fusarium, gene action, heritability, genetics

## Abstract

A comprehensive understanding of the genetics of resistance is essential for developing an effective breeding strategy to create germplasm resistant to Fusarium Ear Rot. This study aimed to determine the general combining ability (GCA), specific combining ability (SCA), and heritability of resistance to infection by *Fusarium verticillioides* in tropical maize. Using the North Carolina II mating design, six inbred lines as females and seven as males were crossed to produce 42 hybrids, which were evaluated across five environments using artificial inoculation. At harvest, the hybrids were scored for Fusarium Ear Rot (FER) infection using a 1-9 severity scale. Significant GCA effects for the parents and SCA effects for the hybrids were observed. The narrow-sense heritability estimate was 0.22, while the broad-sense heritability was 0.73, and the additive genetic effects, as represented by GCA (m+f), were more significant than non-additive effects. The inbred parents JPS25-13, JPS26-125, JPS26-86, JPS25-11, JPS25-5, JPS25-7, and JPS25-9 were identified as the best general combiners for FER resistance. These lines, with favorable general combining ability effects for resistance to *Fusarium verticillioides*, are strong candidates for breeding resistant varieties.

## 1 Introduction

Maize is one of the most important cereal crops globally due to its high yield potential and its widespread use as food, animal feed, and for various industrial applications (Ju et al., 2017). In sub-Saharan Africa, maize serves as a critical source of food security and economic development (Cairns and Prasanna, 2018), with over 300 million Africans relying on it as their primary staple food (Badu-Apraku and Fakorede, 2017). In Uganda, maize is a staple food for many households in both rural and urban areas (Epule et al., 2021), with total production in the 2020 agricultural year estimated at approximately 3.5 million metric tonnes (MT) from around 2.0 million hectares (ha) of planted area (UBOS, 2020).

Despite its importance, maize production faces numerous biotic and abiotic challenges, including Fusarium Ear Rot (FER) caused by *Fusarium verticillioides*. This fungal pathogen is widespread and infects various plant species, including maize, millet, rice, wheat, and sorghum (Xu et al., 2023). In maize, it causes ear, stalk, and root rots (Logrieco et al., 2002) and can lead to yield losses of up to 50% (Ding et al., 2008; Magarini et al., 2024). FER is typically favored by warm and dry conditions, although warm and wet conditions following inoculation at silking can also promote disease development (Afolabi et al., 2007). This fungus poses a major problem not only due to the yield losses it causes but also because it produces fumonisin, a mycotoxin harmful to both humans and animals. In Uganda, studies by Atukwase et al. (2009; 2012) reported occurrence of fumonisin in major maize growing districts. A recent study by Yli-Mattila and Sundheim, (2022) reported that Uganda is among African countries with high levels of fumonisins (FUM) in maize, ranging from 270 to 10,000 µg/kg, exceeding the advisory levels of 2000 µg/kg set by the U.S. Food and Drug Administration (FDA) and regulatory limits of 2000–4000 µg/kg set by the East African Community (EAC) and European Union (EU) (Atukwase et al., 2009; Niyibituronsa et al., 2020; Yli-Mattila and Sundheim, 2022). These high levels of fumonisins identified in Uganda are worrying, therefore awareness and management of this fungus is an important step in controlling human exposure to the toxins. Common practices like the use of insecticides, fungicides, or other agronomic approaches have been reported to be environmentally unfriendly, ineffective and, and they increase the costs of maize production (Lanubile et al., 2017; Ayesiga et al., 2023).

Breeding for resistance is the most economical, effective, and environmentally friendly approach to control Fusarium Ear Rot (FER) and reduce the risk of fumonisin accumulation in maize (Netshifhefhe et al., 2018; Ouko et al., 2020). To develop cultivars resistant to *F. verticillioides*, it is essential to identify and utilize sources with strong resistance. However, most commercially available maize cultivars worldwide lack specific resistance to *F. verticillioides* (Tembo et al., 2022) and the known sources of resistance predominantly originate from temperate regions, where they are poorly adapted to the growing conditions in sub-Saharan Africa and often have limited combining ability for yield (Warburton et al., 2009; Tembo et al., 2022). Several studies have reported genetic variation for resistance to FER (Lanubile et al., 2011; Butrón et al., 2015; Stagnati et al., 2019). Efforts to understand the mechanisms and genetics of resistance to *F. verticillioides* infection in maize have highlighted that resistance to FER is quantitatively inherited and is largely associated with additive genetic effects, along with a significant genotype by environment component (Ding et al., 2008; Reid et al., 2009; Mesterházy et al., 2012; Balconi et al., 2014; Butrón et al., 2015; Maschietto et al., 2015; Tembo et al., 2022). Some studies have reported that resistance to Fusarium Ear Rot involves both additive and nonadditive genetic effects, highlighting the complexity of the trait and the need to consider both types of genetic contributions in breeding programs (Chen et al., 2012; Lanubile et al., 2017; de Jong et al., 2018). However, no complete resistance to FER has been identified in maize (Eller et al., 2008). Mukanga et al. (2010) found high genotype × environment interaction (G × E) for ear rot-causing fungi, implying that different genotypes respond variably to infection across different environments. Tembo et al. (2022) studied twelve inbred lines from Seed Co used as the females mated to 12 tester lines from the International Institute of Tropical Agriculture (IITA) using the North Carolina Design II. The 144 F1 hybrids and six check hybrids were evaluated in Zimbabwe under artificial inoculation with *F. verticillioides*. The GCA and SCA effects for *F. verticillioides* incidence were variable across sites for the lines and the testers. The study identified six southern African inbred lines with desirable GCA for FER and could be used as resistance sources. Both additive and nonadditive effects were implicated in resistance to FER. Rose et al. (2016) evaluated 18 inbred lines for resistance to FER in five locations in South Africa and found significant environment X genotype interactions and also reported additive gene effects were involved in resistance to FER.

Efficient breeding for resistance to FER requires a thorough understanding of its genetic basis; combining ability and heritability are essential factors when studying the genetics of crop traits (Ma Teresa et al., 1994). The objectives of this study were to evaluate the resistance of F1 hybrids to *F. verticillioides* and to determine the mode of gene action conditioning resistance to Fusarium Ear Rot.

## 2 Materials and methods

### 2.1 Plant materials and generation of F1 hybrids

In this study, thirteen inbred lines (**Table 1**) with varying levels of resistance to FER were used to investigate the genetic action behind the resistance to FER. The parents were chosen based on their contrasting responses to FER observed in preliminary screening trials, and their genetic diversity (Ayesiga et al., 2022, 2023), which aimed to maximize the potential for detecting combining ability effects. These parental lines encompass a substantial portion of the breeding material currently used in national programs, making the findings relevant to ongoing breeding programs in the region. The lines are owned and maintained by the National Agricultural Research Organization (NARO), ensuring accessibility for further research and development. All possible crosses between the seven males resistant lines and six females susceptible lines were made using North Carolina II mating design (Comstock and Robinson, 1952) under controlled pollination conditions to produce 42 hybrids. These hybrids were then evaluated in three locations in Uganda as described below.

**Table 1:** List parental lines used and their pedigrees.

Parent	Inbred line	Status	Pedigree
Males			
**1**	JPS26-125	FER resistant	POBLAC2ICO/WL 118-17-4-3-1
**2**	JPS25-22	FER resistant	(NML85/(La Posta Seq C7-F71-1-2-1-2-B-B-B/CML312SR = MAS[MSR/312]-117-2-2-1-2-B*4-B-B-B-B/CML312SR) DH-10-B-B-B)-B-1-2-2-B-B
**3**	JPS25-36	FER resistant	(NML85/(La Posta Seq C7-F71-1-2-1-2-B-B-B/CML312SR = MAS[MSR/312]-117-2-2-1-2-B*4-B-B-B-B/CML312SR) DH-18-B-B-B)-B-2-2-2-B-B
**4**	JPS25-13	FER resistant	(NML85/(La Posta Seq C7-F96-1-2-1-1-B-B-B/CML444/CML444) DH-104-B-B-B)-B-3-1-2-B-B
**5**	JPS26-4	FER resistant	POBLAC21CO/NML 56-1-2-3
**6**	JPS26-86	FER resistant	POBLAC2ICO/NML 89-5-2-2
**7**	JPS25-14	FER resistant	(NML85/(La Posta Seq C7-F96-1-2-1-1-B-B-B/CML444/CML444) DH-104-B-B-B)-B-3-2-1-B-B
Females			
**1**	JPS25-5	FER susceptible	(NML85/(La Posta Seq C7-F96-1-2-1-1-B-B-B/CML444/CML444) DH-104-B-B-B)-B-1-1-5-B
**2**	JPS25-7	FER susceptible	(NML85/(La Posta Seq C7-F96-1-2-1-1-B-B-B/CML444/CML444) DH-104-B-B-B)-B-1-1-8-B
**3**	JPS25-8	FER susceptible	(NML85/(La Posta Seq C7-F96-1-2-1-1-B-B-B/CML444/CML444) DH-104-B-B-B)-B-1-1-10-B
**4**	JPS25-9	FER susceptible	(NML85/(La Posta Seq C7-F96-1-2-1-1-B-B-B/CML444/CML444) DH-104-B-B-B)-B-2-1-1-B
**5**	JPS25-11	FER susceptible	(NML85/(La Posta Seq C7-F96-1-2-1-1-B-B-B/CML444/CML444) DH-104-B-B-B)-B-2-1-4-B-B
**6**	JPS25-12	FER susceptible	(NML85/(La Posta Seq C7-F96-1-2-1-1-B-B-B/CML444/CML444) DH-104-B-B-B)-B-2-1-6-B-B

### 2.2 Description of the study sites

Nakyesasa falls in the mid-altitude agro-ecological zone, located at 0° 32’N and 32° 35´E, at 1150 meters above sea level, the soil type is sandy clay loam and is classified as Orthic Ferrasol. The mean annual rainfall at Nakyesasa is 1270 mm with a bimodal distribution (March–July and September–November). Buginyanya is located at 1°12’48.0”N and 34°23’35.0”E, at 1877 meters above sea level and is characterized with well drained dark clay loam and red sand clay loam with a sticky texture and is classified as Plinthic Umbric. The region receives a bi-modal pattern of rainfall, with the wettest months being April and October and the annual average rainfall in the zone ranges from 1,200mm to 2,000mm. Bulindi is located at 0°16’N, 32^0^52’E’, at 1144 meters above sea level, the soil is Sandy loam and is classified as Acric Ferralsol. The mean annual rainfall at Bulindi is 1338 mm, with a bimodal distribution (March–July and September–November).

### 2.3 Field evaluation

Forty-two single-cross hybrids were evaluated in this trial, conducted across three sites (Nakyesasa, Buginyanya, and Bulindi) over two growing seasons (A and B). Uganda has two seasons; season A which runs from February to June and season B runs from September to December. Buginyanya season A (E1) was planted on 22^nd^/04/2022; Nakyesasa season A (E2) was planted on 14^th^/04/2022; Nakyesasa season B (E3) was planted on 7^th^/09/2022; Buginyanya season B (E4) was planted on 30^th^/09/2022 and Bulindi season B (E5) was planted on 24^th^/09/2022. The experiment followed a 6 × 7 alpha lattice design with two replications at each location. Each hybrid was planted in two-row plots measuring 5 meters in length, with rows spaced 0.75 meters apart and 0.25 meters between plants. Two seeds were sown per hill, and after four weeks, the seedlings were thinned to one plant per hill. Standard agronomic practices, including weeding and appropriate fertilizer applications, were applied consistently across all trials. Although cycle length was not directly evaluated in this study, artificial inoculations were standardized at seven days after flowering to ensure consistency in FER resistance evaluation across hybrids following the technique outlined by Ayesiga et al. (2023), in brief, fully colonized toothpicks were used to inoculate the maize ears approximately seven days after flowering. Inoculation was conducted by piercing through the middle of the primary ear of five plants per plot. Paper bags were used to cover the ears to avoid allo-infection. At maturity, the inoculated ears from each plot were harvested, and FER symptoms were assessed based on the percentage of infected area using the following nine-point scale: 1 = 0% (no visible disease symptom), 2 = 1%, 3 = 2%–5%, 4 = 6%–10%, 5 = 11%–20%, 6 = 21%–40%, 7 = 41%–60%, 8 = 61%–80%, and 9 = 81%–100% (**Supplementary Figure S1**). Scores of 1–2 were classified as “good,” 3–5 as “intermediate,” and 6–9 as “poor.” (Tembo et al., 2022).

### 2.4 Data analysis

#### 2.4.1 Analysis of variance

The data was subjected to Analyses of variance in R software using the agricolae package (De Mendiburu, 2021) in R software (R Core Team, 2021),. Each location-season combination was considered an environment. Environments and replications were treated as fixed effects and the other effects as random. Across location linear model:


$$\begin{array}{c} Y_{ijk}=\mu +{\text{genotype}}_{i}+{\text{Env}}_{j}+{\left(\text{genotype}\times \text{Env}\right)}_{ij}+\text{rep}{\left(\text{Env}\right)}_{jk}\\ +{\text{error}}_{ijk}; \end{array}$$

where Y*ijk* is the phenotypic value of the *i*th genotype, *j*th environment, *k*th replication, *jk*, the effect of *k*th replication in *j*th environment and error*ijk* is the residual.

Variance components were calculated using the linear mixed model:


Yijko=μ+gi+lj+rkj+bojk+eijko,

where *Yijko* is the phenotypic value of the *i*th genotype at the *j*th environment in the *k*th replication of the *o*th incomplete block, *gi* is the effect of the *i*th genotype, *lj*, the effect of the *j*th environment, *rkj*, the effect of the *k*th replication at the *j*th environment, *bojk* is the effect of the *o*th incomplete block in the *k*th replication at the *j*th environment, and *eijko* is the residual.

The Tukey’s HSD *post-hoc* test was performed to determine significant differences among the means of genotypes across environments. This test provides pairwise comparisons while controlling the family-wise error rate. The analysis was conducted using the agricolae package (De Mendiburu, 2021) in R software (R Core Team, 2021).

#### 2.4.2 Determination of combining ability

The General Combining Ability (GCA) effects for each parent were analyzed, as well as the Specific combining ability (SCA) effects for the hybrids were Analyzed with R for Windows (AGD-R) (Rodríguez et al., 2015) software using the Henderson method to estimate variances using the model;


$$\begin{array}{c} Yijkq=\mu +gi+gj+sij+yq+rk(yq)+(gy)iq+(gy)jq+(sy)ijq\\ +eijkq \end{array}$$

where *i* = 1, 2, …., 6; *j* = 1, 2, …., 7; *k* = 1, 2; *q* = 1, 2, 3, 4, 5; and *Yijkq* is the value of the hybrid from mating the *j*th male line and the *i*th female line in the *k*th replication, in the *q*th environment; μ is the grand mean, *gi* is the GCA effect for the progeny of the *i*th female line, *gj* the general combining ability effect for the progeny of the *j*th male line, *sij* the SCA effect for hybrid got from mating the *i*th female line and the *j*th male line, *yq* is the average effect of the *q*th environment, *rk*(*yq*) is the effect of the *k*th replication nested within the *q*th environment, (*gy*)*iq* and (*gy*)*jq* are the interactions between the GCA effects and the environment, (*sy*)*ijq* is the interaction between the SCA effect and environment, and *eijkq* is the residual.

Broad sense (H^2^) and Narrow Sense (h^2^) heritabilities were also estimated respectively using the formulae by (Falconer and Mackay, 1996);


$$h^{2}=\frac{2{\text{δ}}^{2}GCA}{2{\text{δ}}^{2}GCA+{\text{δ}}^{2}SCA+{\text{δ}}^{2}e}$$

Where; *h^2^* = narrow sense heritability; δ^2^ *GCA* = variance of general combining ability; δ^2^ *SCA* = variance of specific combining ability of parents; δ^2^ *e* = error variance.


$$H^{2}=\;\frac{\sigma_{g}^{2}}{\sigma_{g}^{2}+\;\frac{\sigma_{g\;\times e}^{2}}{n_{e}}+\;\frac{\sigma_{e}^{2}}{n_{e}n_{r}}}$$

Where;

*H^2^* = broad sense heritability;

$\sigma_{g}^{2}$ is the genetic variance,

$\sigma_{g\;\times e}^{2}$ is the genotype-by-environment interaction variance,

$\sigma_{e}^{2}$ is the residual error variance

$n_{e}$ is the number of environments,

$n_{r}$ is the number of replications.

The predominant gene action in the inheritance of resistance to FER was estimated using Baker’s ratio (BR) (Baker, 1978), computed as;


$$BR=\frac{\left(2*{\text{δ}}^{2}GCA\right)}{\left(2{\text{*δ}}^{2}GCA+{\text{δ}}^{2}SCA\right)}$$

## 3 Results

### 3.1 Mean performance of the maize hybrids for FER across five test environments

**Table 2** presents the performance of 42 different hybrids (labeled JPSH-1 to JPSH-42) across five environments (E1 to E5) and their corresponding mean values. The means ranged from 2.9 to 6.9, with an overall mean of 4.6 across the five environments. The hybrids exhibited different performances across environments, with some performing consistently well or poorly across all environments, while others show more variability. The hybrids with the most outstanding performance for FER resistance were JPSH-3, JPSH-35, JPSH-4, JPSH-1, JPSH-5, JPSH-12, JPSH-16, JPSH-23, JPSH-29, JPSH-32 and JPSH-33 with mean severity scores less than 4.0. The most susceptible hybrids with mean severity scores above 5.0 were JPSH-18, JPSH-15, JPSH-30, JPSH-36, JPSH-42, JPSH-10, JPSH-11, JPSH-13, JPSH-20, JPSH-21 and JPSH-22. The mean scores for each environment (E1 to E5) varied, suggesting that environmental conditions significantly influence hybrid performance. Based on the studied environments, the lowest FER severity score was recorded in E2 followed by E5 and the highest score was in E4. **Table 2** also presents the CV which is the measure of relative variability, expressed as a percentage, a higher CV suggests more variability in the data. Environments E2 and E5 had the highest CV values (59% and 59.8%, respectively), suggesting more variability in hybrid performance in those environments compared to E4, which has a lower CV (20.4%). Differences in hybrid performance across environments may be influenced by traits such as cycle length, which was not assessed in this study. Future evaluations could explore the relationship between cycle length and resistance to Fusarium Ear Rot. Also, while yield data were not collected, visual observations suggested varying levels of plant vigor and ear development among hybrids, warranting further investigation into their yield potential.

**Table 2:** Mean Fusarium Ear Rot severities for 42 maize hybrids assessed in five test environments within Uganda.

Hybrid	Designation	E1	E2	E3	E4	E5	Mean
JPSH1	JPS26-125JPS25-5	4	2.5	4	5	1.5	3.4
JPSH2	JPS26-125/JPS25-7	3.5	3	3.5	7	4	4.2
JPSH3	JPS26-125/JPS25-8	2	2	2.5	6.5	1.5	**2.9**
JPSH4	JPS26-125/JPS25-9	3.5	3	4	4	1	**3.1**
JPSH5	JPS26-125/JPS25-11	2.5	2	2.5	8.5	2.5	3.6
JPSH6	JPS26-125/JPS25-12	5	2.5	7.5	3	5	4.6
JPSH7	JPS25-22/JPS25-5	6	3.5	5	7.5	2.5	4.9
JPSH8	JPS25-22/JPS25-7	6	3.5	4	8.5	2.5	4.9
JPSH9	JPS25-22/JPS25-8	6	2.5	5	5	6	4.9
JPSH10	JPS25-22/JPS25-9	7	1	5	7.5	5	5.1
JPSH11	JPS25-22/JPS25-11	4	5.5	3	8.5	5	5.2
JPSH12	JPS25-22/JPS25-12	3	2.5	3	8	2.5	3.8
JPSH13	JPS25-36/JPS25-5	9	1	2	8.5	6	5.3
JPSH14	JPS25-36/JPS25-7	5.5	1.5	5	4.5	3.5	4
JPSH15	JPS25-36/JPS25-8	6	8	4.5	6.5	8.5	6.7
JPSH16	JPS25-36/JPS25-9	4.5	3	4.5	5	2.5	3.9
JPSH17	JPS25-36/JPS25-11	3.5	4.5	6	2	2	3.6
JPSH18	JPS25-36/JPS25-12	8	5	7	7.5	7	6.9
JPSH19	JPS25-13/JPS25-5	5.5	3	6	2	1.5	3.6
JPSH20	JPS25-13/JPS25-7	7.5	3	5	9	2.5	5.4
JPSH21	JPS25-13/JPS25-8	4.5	3.5	7.5	6.5	4.5	5.3
JPSH22	JPS25-13/JPS25-9	6	3	6	7.5	3.5	5.2
JPSH23	JPS25-13/JPS25-11	6	2.5	2.5	2.5	1.5	**3**
JPSH24	JPS25-13/JPS25-12	7	4	3.5	8.5	4.5	5.5
JPSH25	JPS26-4/JPS25-5	7	2.5	6.5	5.5	4	5.1
JPSH26	JPS26-4/JPS25-7	3	3	4	6.5	7	4.7
JPSH27	JPS26-4/JPS25-8	5.5	2.5	4.5	5	6.5	4.8
JPSH28	JPS26-4/JPS25-9	2	5	7	1.5	6	4.3
JPSH29	JPS26-4/JPS25-11	3	2.5	5	3.5	1.5	**3.1**
JPSH30	JPS26-4/JPS25-30	8.5	2	7	7.5	6	6.2
JPSH31	JPS26-86/JPS25-5	6	2	3	5	4	4
JPSH32	JPS26-86/JPS25-7	3	3.5	3	7.5	2	3.8
JPSH33	JPS26-86/JPS25-8	4.5	3	3	5.5	3	3.8
JPSH34	JPS26-86/JPS25-9	5	3.5	7	8	5.5	5.8
JPSH35	JPS26-86/JPS25-11	2.5	1.5	2.5	7	1.5	**3**
JPSH36	JPS26-86/JPS25-12	5	5	3	9	8	6
JPSH37	JPS25-14/JPS25-5	6.5	3	6.5	8	3	5.4
JPSH38	JPS25-14/JPS25-7	7	4	6	7.5	1	5.1
JPSH39	JPS25-14/JPS25-8	4.5	3	7	6	4.5	5
JPSH40	JPS25-14/JPS25-9	3.5	2.5	5.5	8.5	2	4.4
JPSH41	JPS25-14/JPS25-11	8.5	5.5	5.5	7.5	1.5	5.7
JPSH42	JPS25-14/JPS25-12	5	2	7.5	8.5	7	6
	Mean	5.1	**3.1**	5	6.1	3.8	4.6
	LSD	1.8	1.3	1.7	2.2	2.1	1
	CV	47.6	59	44.4	20.4	59.8	0.22

E1 Buginyanya2022A; E2 Nakyesasa2022A; E3 Nakyesasa2022B; E4 Buginyanya2022B; E5 Bulindi2022B; LSD Least significant difference; CV Coefficient of variation. In bold: Lowest mean values indicating resistance.

### 3.2 ANOVA of the FER severity for the hybrids across five environments

The results of Analysis of variance are shown in **Table 3**, and varying degrees of *F. verticillioides* infection were observed across test environments. The combined analysis of variance across the five environments showed that environment and genotypes were highly significant (*P* < 0.001) for kernel infection, while the Genotype × Environment interaction was significant at *P* < 0.05. The F1 hybrid mean square was partitioned into male general combining ability (GCA_MALE_), female general combining ability (GCA_FEMALE_), and specific combining ability components. The mean squares for (GCA_MALE_) and (GCA_FEMALE_) were highly significant (*P* < 0.001). The mean squares for GCA_MALE_ × environment and GCA_FEMALE_ × Environment interaction effects were highly significant (*P* < 0.001) and significant at *P* < 0.05 respectively. The ANOVA results (**Table 3**) indicate that the overall contributions of both male and female GCA effects are significant, reflecting the importance of additive genetic effects for resistance to Fusarium Ear Rot. However, when examining individual lines in **Table 4**, JPS26-125 was the only male line with a statistically significant negative GCA effect, while other male and female lines also contributed to resistance but did not reach statistical significance individually.

**Table 3:** Mean squares and degrees of freedom for the combined analysis of variance for percentage kernel infection for 42 tropical maize hybrids in 5 environments of Uganda.

Source	Df	Mean Sq
Environment	4	78.30***
Rep(Env)	5	2.09***
Genotypes	41	10.00***
GCA_MALE_	6	16.14***
GCA_FEMALE_	5	17.85***
SCA	30	7.62*
Genotype × Env	164	5.72*
GCA_MALE_ × Env	24	8.47***
GCA_FEMALE_ × Env	20	7.76*
SCA × Env	120	4.79^ns^
Residuals	155	4.36
Baker’s Ratio		0.67
Broad Sense Heritability		0.73
Narrow Sense Heritability		0.22

Df Degrees of freedom; FER Fusarium Ear Rot; (%); level of significance ***(1%), **(5%), *(10%); ns, not significant; GCA_MALE_ male general combining ability, GCA_FEMALE_, female general combining ability; SCA, specific combining ability.

**Table 4:** General combining ability effects of (GCAFEMALE) and (GCAMALE) for FER infection across the five environments.

	GCA	Rank
Males		
JPS25-13	-0.03	3
JPS25-14	0.61	7
JPS25-22	0.18	6
JPS25-36	0.41	5
JPS26-125	-0.98*	1
JPS26-4	0.11	4
JPS26-86	-0.30	2
Females		
JPS25-11	-0.75	1
JPS25-12	0.86	6
JPS25-5	-0.09	3
JPS25-7	-0.02	4
JPS25-8	0.11	5
JPS25-9	-0.13	2

### 3.3 Analysis of general combining ability for *Fusarium verticillioides* ear rot

The results for the combining ability of the 13 parents for FER severity are presented in **Table 4**. Based on GCA effects, the best inbred parents for producing hybrids with resistance to FER were those with negative GCA. Male inbred lines JPS26-125, JPS26-86 and JPS25-13, with JPS26-125 having the most negative (-0.98) GCA while JPS25-14 had the most positive effect of 0.61. Among the female inbred lines, JPS25-11 exhibited the most negative GCA effect (-0.75), indicating its strong contribution to resistance to Fusarium Ear Rot (**Table 4**).

### 3.4 Analysis of specific combining ability *Fusarium verticillioides* ear rot

The results of the SCA of the crosses are presented in **Table 5**. The Specific Combining Ability effects for FER were significant at *P* < 0.05, whereas the SCA x environment interaction was not significant across the five locations. Out of the 42 hybrids, 21 had negative SCA values but only three were significant, these being JPS25-11//JPS25-13, JPS25-12//JPS25-22 and JPS25-11//JPS26-4, thus considered the best combinations. Hybrid JPS25-12//JPS25-22 had the most negative (-1.97) SCA effects for *F. verticillioides* ear rot across the environments and it was significant, whereas the highest positive value was found for hybrid JPS 25-9/JPS26-86.

**Table 5:** Estimates of specific combining ability effects for FER scores of 13 inbred lines across the environments.

FEMALE	MALE	MALE × FEMALE MEAN	SCA	RANK
JPS25-11	JPS25-13	2.96*	-0.91	5
JPS25-12	JPS25-13	5.32	-0.17	18
JPS25-5	JPS25-13	3.55	-0.97	3
JPS25-7	JPS25-13	5.45	0.85	38
JPS25-8	JPS25-13	5.28	0.56	31
JPS25-9	JPS25-13	5.12	0.63	33
JPS25-11	JPS25-14	5.67	1.16	40
JPS25-12	JPS25-14	6.05	-0.08	19
JPS25-5	JPS25-14	5.41	0.25	27
JPS25-7	JPS25-14	5.2	-0.04	20
JPS25-8	JPS25-14	4.93	-0.44	13
JPS25-9	JPS25-14	4.28	-0.86	6
JPS25-11	JPS25-22	5.16	1.08	39
JPS25-12	JPS25-22	3.74**	-1.97	1
JPS25-5	JPS25-22	4.97	0.23	26
JPS25-7	JPS25-22	4.95	0.14	25
JPS25-8	JPS25-22	4.97	0.03	22
JPS25-9	JPS25-22	5.19	0.49	29
JPS25-11	JPS25-36	3.65	-0.66	10
JPS25-12	JPS25-36	6.73	0.8	37
JPS25-5	JPS25-36	5.36	0.4	28
JPS25-7	JPS25-36	4.06	-0.98	2
JPS25-8	JPS25-36	6.58	1.41	41
JPS25-9	JPS25-36	3.97	-0.97	4
JPS25-11	JPS26-125	3.7	0.78	36
JPS25-12	JPS26-125	4.59	0.04	24
JPS25-5	JPS26-125	3.4	-0.17	17
JPS25-7	JPS26-125	4.25	0.6	32
JPS25-8	JPS26-125	2.95	-0.83	8
JPS25-9	JPS26-125	3.12	-0.42	14
JPS25-11	JPS26-4	3.17*	-0.85	7
JPS25-12	JPS26-4	6.33	0.69	35
JPS25-5	JPS26-4	5.16	0.49	30
JPS25-7	JPS26-4	4.78	0.03	23
JPS25-8	JPS26-4	4.85	-0.02	21
JPS25-9	JPS26-4	4.29	-0.35	15
JPS25-11	JPS26-86	2.99	-0.6	12
JPS25-12	JPS26-86	5.9	0.69	34
JPS25-5	JPS26-86	4.02	-0.23	16
JPS25-7	JPS26-86	3.71	-0.61	11
JPS25-8	JPS26-86	3.74	-0.71	9
JPS25-9	JPS26-86	5.69	1.47	42

Level of significance; **(5%), *(10%).

## 4 Discussion

It is important to note that the study involved a small sample of seven males and six females selected for their FER resistance. As such, the conclusions drawn here pertain specifically to the genotypes evaluated and should not be generalized to the broader maize germplasm, including Ugandan maize populations. Never the less, improving maize yield is a key priority for breeders to meet the rising global demand, especially by managing diseases like FER. Breeding for resistance is the most effective strategy for controlling FER, particularly for smallholder farmers who primarily grow maize for subsistence and often lack the resources to implement other control measures (Chen et al., 2016). However, to optimize this approach, it is crucial to identify stable and effective sources of resistance and understand their performance across different environments. This is especially important since FER resistance is a complex trait, controlled by many genes with minor individual effects, exhibiting low to moderate heritability, and highly influenced by environmental factors.

In this study, significant variation was observed among the hybrids (P < 0.001), indicating substantial genetic diversity that could facilitate selection progress for FER resistance under artificial inoculation. This variation is attributed to the hybrids’ differing responses to the fungus and the distinct resistance mechanisms employed (Hung and Holland, 2012). The presence of significant variation among inbred lines and their hybrids for *Fusarium verticillioides* infection offers an opportunity for genetic improvement in local breeding programs (Clements et al., 2004; Tembo et al., 2022). Moreover, the significant genotype-by-environment interactions (P < 0.1) suggest that the hybrids performed differently across the study locations, highlighting the role of environmental factors in their differential responses (Tembo et al., 2022). This underscores the importance of testing hybrids across multiple environments to select those suited to specific production areas or with broad adaptation potential (Mugisa et al., 2022). Similar findings were reported by Ouko et al. (2020); Tembo et al. (2022), and Netshifhefhe et al. (2018). The observed genotype × environment interactions in this study could be influenced by unmeasured traits such as cycle length, which has been correlated with rotting disease resistance in previous studies (Agrama and Moussa, 1996; Zwonitzer et al., 2010; Chabanne et al., 2020). Incorporating this trait in future evaluations could provide a more comprehensive understanding of FER resistance.

Partitioning the general combining ability (GCA) into male and female components revealed highly significant GCA effects, indicating that additive genetic effects from both male and female parents are crucial. The specific combining ability (SCA) was marginally significant (P < 0.05), suggesting that non-additive genetic effects also play a role, albeit making a smaller contribution. Similar findings were reported by various studies using different materials and in different environments among them; Tembo et al. (2022) and Ouko et al. (2020).

In this study, negative combining ability effects were desirable, as they indicate a line’s contribution to resistance, while positive combining ability effects suggest susceptibility (Mukanga et al., 2010; Tembo et al., 2022). Based on GCA effects, the best inbred parents for producing FER-resistant hybrids were JPS25-13, JPS26-125, JPS26-86, JPS25-11, JPS25-5, JPS25-7, and JPS25-9. Incorporating these resistant lines into breeding programs could strengthen resistance to FER in future hybrids (Ouko et al., 2020). These lines could be used in recurrent selection programs or as testers in hybrid combinations. The significant contribution of lines with high GCA effects highlights the predominance of additive gene action in conferring resistance to FER. Although specific hybrids displayed significant SCA effects, these were limited and represented exceptions rather than the majority of the tested hybrids. The hybrids JPS25-12/JPS25-22, JPS25-11//JPS26-4, and JPS25-11/JPS25-13 stand out for their significant negative SCA effects, showing strong non-additive contributions to resistance. These crosses could serve as models for generating high-performing hybrids in different environments.

**Figure 1:**
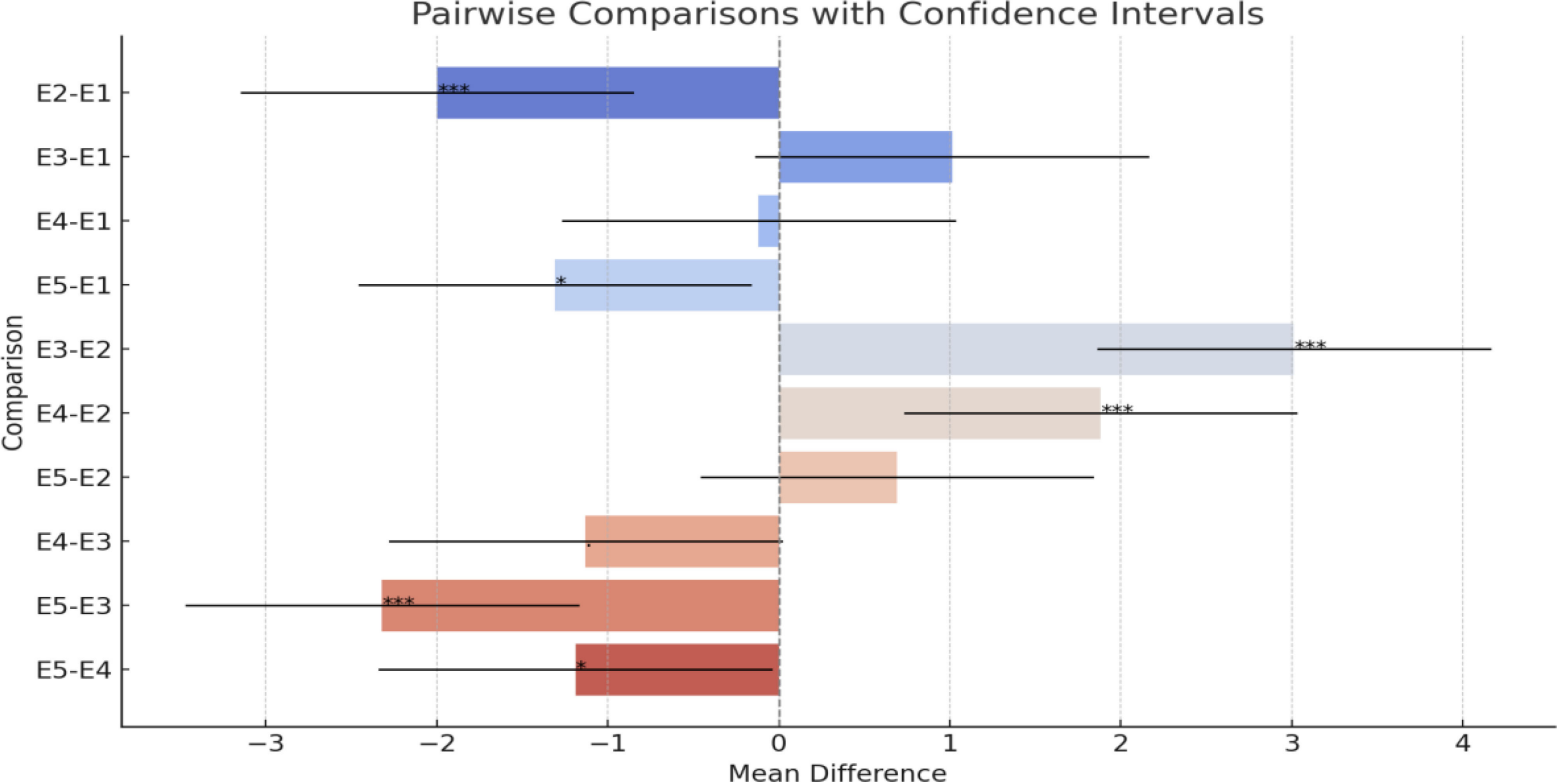
Representation of the pairwise comparisons with their confidence intervals: • The bars represent the mean differences between environments. • The horizontal lines extending from each bar indicate the confidence intervals (95%). • The dashed vertical line at 0 highlights where no difference exists. • The annotations next to the bars indicate the significance levels (***, *,., or none).

The environment significantly influenced the observed variation in fungal colonization, as shown in **Table 2**, with differing conditions favoring the spread of *F. verticillioides*. This was evident in the differences in the hybrids’ responses between the five environments. For example, hybrid JPSH-13 exhibited contrasting responses, being susceptible in environments E1, E4, and E5, but resistant in E2 and E3, a pattern seen in several hybrids environment E4 (Buginyanya) had the highest infection scores, likely due to high relative humidity and rainfall, which promote the fungus’s growth and spread. The same result was also observed by Ouko et al. (2020). The significant GCA × environment interactions in this study suggest that selecting parental lines tailored to specific environments could enhance resistance (Mukanga et al., 2010). A visual representation in **Figure 1** of pairwise comparisons for Ear Rot among the different environments highlights significant differences in mean values. Notably, the comparison between E2 and E1 shows a highly significant negative difference, indicating that E2 has a considerably lower mean Ear Rot than E1. Similarly, E3 exhibits a significantly higher mean Ear Rot compared to E2, as evidenced by the positive difference and non-overlapping confidence intervals. Other comparisons, such as E5-E1 and E5-E3, reveal significant differences, with E5 generally exhibiting lower mean values. In contrast, some comparisons, like E3-E1 and E4-E1, show no statistical significance, as their confidence intervals include zero, suggesting similar performance between these environments. Overall, the results demonstrate notable variability in Ear Rot severity across environments, with certain environments performing significantly better or worse than others. These findings underscore the importance of environment-specific factors in influencing Ear Rot levels.

The broad-sense heritability in this study was relatively high (0.73), indicating that a large portion of the phenotypic variance was attributable to genetic factors such as additive, dominance, and epistatic effects. This suggests that over 70% of the observed phenotypic variance in the hybrids was inherited from the parents, reflecting similarities between the inbred lines and their hybrids (Netshifhefhe et al., 2018). The high heritability estimates imply that resistance to FER can be effectively achieved by crossing two parental inbred lines with favorable GCA effects (i.e., negative GCA for disease resistance). The strong heritability of resistance to *Fusarium verticillioides* suggests that resistance levels in the hybrids closely resemble those of their parent inbred lines (Netshifhefhe et al., 2018), making phenotypic selection for improved resistance to FER a viable strategy. This information can therefore guide breeding programs in developing maize hybrids with enhanced resistance to FER. The Baker’s ratio of 0.67 indicates that additive genetic effects, as captured by the GCA, play a more significant role than non-additive effects (SCA) in the inheritance of resistance to FER. This aligns with the ANOVA results, which show highly significant GCA effects for both male and female parents, and these results are consistent with findings by Rose et al. (2016) and Hung and Holland (2012).

The findings of this study are useful to aid breeders in selecting maize lines with resistance to mycotoxigenic fungi for the development of hybrids with improved tolerance to mycotoxin contamination in Uganda and other tropical countries. Additive gene effects were important in conferring resistance to and ear rot. Furthermore, hybrids with improved resistance to *F. verticillioides* infection were generated, and parental lines served as a good indicator of a hybrid’s performance to infection. Although this study focused on FER resistance, yield is a critical factor for farmer adoption. Future evaluations of these hybrids should prioritize identifying combinations that balance resistance with high productivity, ensuring both economic viability and disease control. Future research should incorporate the evaluation of cycle length and yield potential to ensure the development of hybrids that combine Fusarium Ear Rot resistance with superior agronomic performance.

## Data Availability

The original contributions presented in the study are included in the article/**Supplementary Material**. Further inquiries can be directed to the corresponding author.

## Supplementary material

Supplementary Figure 1 FER scores 1-9 as scored in the field at harvest.
