# Supplementary figures and images for: Combining ability and gene action for resistance to Fusarium ear rot in tropical maize hybrids
Source: Front Plant Sci. 2025 Jan 30;16:1509859. doi: 10.3389/fpls.2025.1509859 (PMC11821605; doi:10.3389/fpls.2025.1509859)

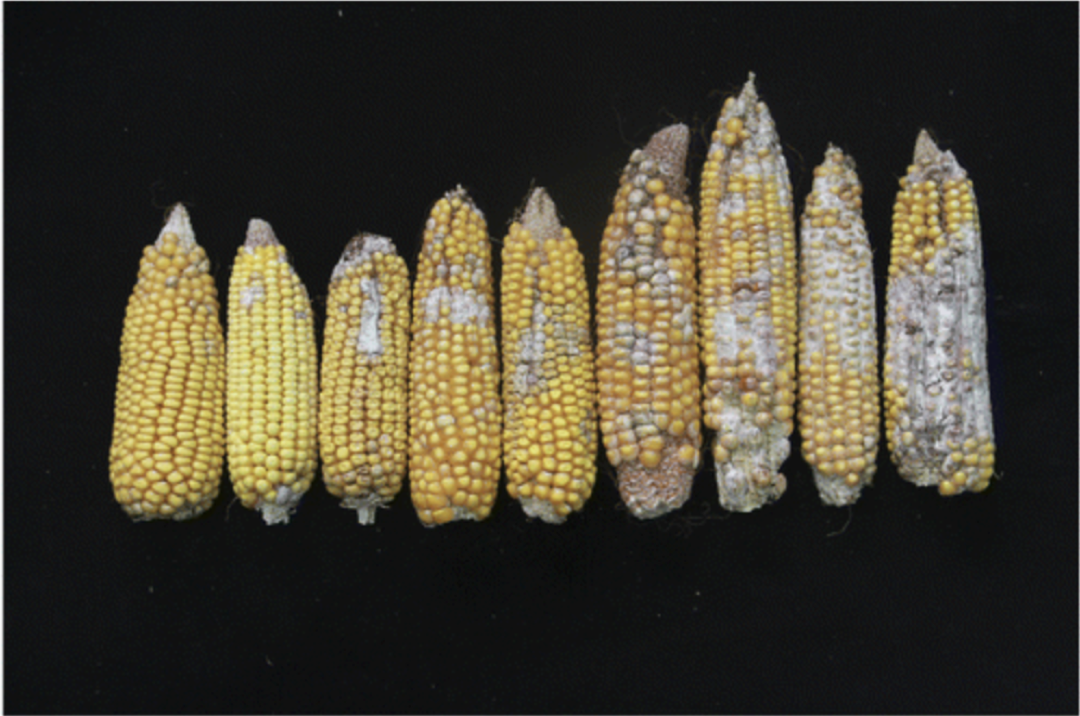


8

9

7

6

5

4

3

2

1

Figure S1: FER scores 1-9 as scored in the field at harvest

Supplement: Supplementary Figure 1 — FER scores 1-9 as scored in the field at harvest. [file DataSheet1.docx]
